# Anti-*Candida* Activity of *Bursera morelensis* Ramirez Essential Oil and Two Compounds, α-Pinene and γ-Terpinene—An In Vitro Study

**DOI:** 10.3390/molecules22122095

**Published:** 2017-12-05

**Authors:** C. Rebeca Rivera-Yañez, L. Ignacio Terrazas, Manuel Jimenez-Estrada, Jorge E. Campos, Cesar M. Flores-Ortiz, Luis B. Hernandez, Tonatiuh Cruz-Sanchez, German I. Garrido-Fariña, Marco A. Rodriguez-Monroy, M. Margarita Canales-Martinez

**Affiliations:** 1 Laboratorio de Farmacognosia, Unidad de Biología, Tecnología y Prototipos (UBIPRO), Facultad de Estudios Superiores Iztacala, Universidad Nacional Autónoma de México, Av. de los Barrios No. 1, Los Reyes Iztacala, Tlalnepantla, Edo. de México C.P. 54090, Mexico; rbkrivera14@gmail.com; 2 Unidad de Biomedicina, Facultad de Estudios Superiores Iztacala, Universidad Nacional Autónoma de México. Av. de los Barrios No. 1, Los Reyes Iztacala, Tlalnepantla, Edo. de México C.P. 54090, Mexico; literrazas@campus.iztacala.unam.mx; 3 Instituto de Química, Universidad Nacional Autónoma de México, Circuito Exterior, Ciudad Universitaria, Coyoacán CDMX 04510, Mexico; manuelj@unam.mx; 4 Laboratorio de Bioquímica Molecular, Unidad de Biología, Tecnología y Prototipos (UBIPRO), Facultad de Estudios Superiores Iztacala, Universidad Nacional Autónoma de México. Av. de los Barrios No. 1, Los Reyes Iztacala, Tlalnepantla, Edo. de México C.P. 54090, Mexico; jcampos@unam.mx; 5 Laboratorio de Fisiología Vegetal, UBIPRO y Laboratorio Nacional en Salud, Facultad de Estudios Superiores-Iztacala UNAM. Av. de los Barrios No. 1, Los Reyes Iztacala, Tlalnepantla, Edo. de México C.P. 54090, Mexico; cmflores@unam.mx (C.M.F.-O.); lbarbo@gmail.com (L.B.H.); 6 Laboratorio de Microbiología, Unidad de Investigación Multidisciplinaria, Facultad de Estudios Superiores Cuautitlán, Campo 4, Universidad Nacional Autónoma de México, Carretera Cuautitlán-Teoloyucan Km 2.5, San Sebastián Xhala, Cuautitlán Izcalli, Edo. de México C.P. 54700, Mexico; tonatiuh86@hotmail.com; 7 Laboratorio de Apoyo a Histología y Biología, Departamento de Ciencias Biológicas, Facultad de Estudios Superiores Cuautitlán, Campo 4, Universidad Nacional Autónoma de México. Carretera Cuautitlán-Teoloyucan Km 2.5, San Sebastián Xhala, Cuautitlán Izcalli, Edo. de México C.P. 54700, Mexico; isaurogafa@yahoo.com.mx; 8 Laboratorio de Inmunobiología, Carrera de Medicina, Facultad de Estudios Superiores Iztacala, Universidad Nacional Autónoma de México, Avenida de los Barrios Numero 1, Colonia Los Reyes Iztacala, Tlalnepantla, Edo. de México C.P. 54090, Mexico; dr.marcorodriguez@gmail.com

**Keywords:** *Bursera morelensis*, burseraceae, *Candida albicans*, α-pinene, γ-terpinene

## Abstract

The candidiasis caused by *C. albicans* is a public health problem. The abuse of antifungals has contributed to the development of resistance. *B. morelensis* has demonstrated antibacterial and antifungal activities. In this work the activity of the essential oil of *B. morelensis* was evaluated and for its two pure compounds with analysis of the different mechanisms of pathogenesis important for *C. albicans*. The essential oil was obtained by the hydro-distillation method and analyzed using GC–MS. The anti-*Candida* activity was compared between to essential oil, α-Pinene and γ-Terpinene. GC–MS of the essential oil demonstrated the presence of 13 compounds. The essential oil showed antifungal activity against four *C. albicans* strains. The most sensitive strain was *C. albicans* 14065 (MFC 2.0 mg/mL and MIC_50_ 0.125 mg/mL) with α-Pinene and γ-Terpinene having MFCs of 4.0 and 16.0 mg/mL respectively. The essential oil inhibited the growth of the germ tube in 87.94% (8.0 mg/mL). Furthermore, it was observed that the essential oil diminishes the transcription of the gene INT1. This work provides evidence that confirms the anti-*Candida* activity of the *B. morelensis* essential oil and its effect on the growth of the germ tube and transcription of the gene INT1.

## 1. Introduction

Fungal infections have become increasingly common and represent a growing health problem in patients with weakened immune systems [1,2]. *Candida albicans* is a polymorphic organism which undergoes morphologic transitions between yeast, pseudohyphal and elongated hyphal forms [3]. This yeast is a commensal organism that normally colonizes the mucosal surfaces of healthy individuals and, under conditions of weak host inmmune system, these can become opportunistic pathogens [4,5]. Despite appropriate therapy, mortality resulting from systemic *C. albicans* infection in immunocompromised has reached 30% [6].

The establishment of *C. albicans* infection implies several mechanisms of pathogenicity, such as dimorphism, germ tube growth, expression of different adhesins like INT1p. It is important to emphasize that INT1 gene, has bifunctional properties, contributing to filamentous growth and also encodes an adhesin function [6,7].

In recent years, the incidence of fungal infections especially *Candida*-related disorders termed generally as “candidiasis” has increased dramatically in many countries [8]. On the other hand, it is known that disseminated infections with *C. albicans* cause significant morbidity and mortality among immunocompromised individuals, such as Human Immunodeficiency Virus (HIV) patients, transplant recipients, and cancer patients [8,9,10,11]. Furthermore, progression of the yeast with increasing the resistance of therapeutic conventional medicine increased the need for the most effective treatment [5].

For this reason, medicinal plants are an alternative, that involves discovery of new compounds that can inhibit the growth of this fungal species. The medicinal plants are the primary alternative in Mexican traditional medicine. The Cañada Region located in the Mexican State of Oaxaca, has a great variety of endemics plant which are commonly used in traditional medicine [12]. *Bursera morelensis* Ramírez belongs to the Burseraceae family, which has been used for preparing resin and bark infusions for the treatment of skin wounds [13,14], addition to alleviate diverse pains and bruises [15]. The aim of this study was to evaluate the anti-*Candida* activity of the essential oil extracted from *B. morelensis* and compare the activity of two present compounds in the essential oil.

## 2. Results

The physicochemical data of the essential oil included a density of 0.82 g/mL at 25° C, while 0.154% (*v*/*w*) of oil from the weight of the fresh product was obtained.

As shown in Table 1, thirteen compounds of the essential oil of *Bursera morelensis* were identified by GC–MS analysis which represented 100% of the total composition. The main compounds, which constituted a total of 75% of the peak area were the terpenes, namely γ-Terpinene (65.46%), β-Pellandrene (18.27%), α-Caryophyllene (5.13%) and α-Pinene (2.85%) (Table 1).

### Anti-Candida Assay

The results of the anti-*Candida* activity of the essential oil and pure compounds are shown in Table 2. All strains of *C. albicans* were sensitive when exposed to essential oil. Nevertheless, when comparing the areas of inhibition of the pure compounds it is possible to observe that γ-Terpinene was significantly different from α-Pinene and essential oil (*p* < 0.05) for all strains. Furthermore, there were significant differences between α-Pinene and essential oil in the strain CDBB-L-1003, (*p* < 0.05).

The essential oil showed a Minimum Fungicidal Concentration (MFC) of 2 mg/mL, which is a smaller concentration compared to that obtained by the α-Pinene and γ-Terpinene (4 and 16 mg/mL respectively) (Table 3).

**Figure 1:**
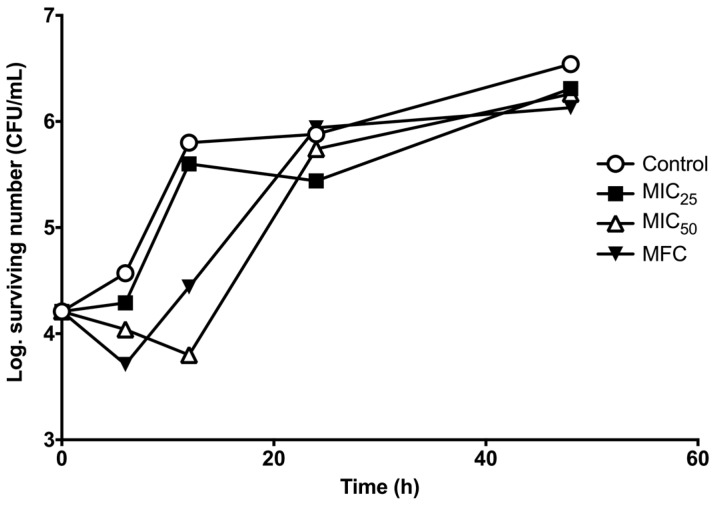
Survival curve of *C. albicans* 14065 after being exposed to essential oil of *B. morelensis*. The essential oil was added to each experimental culture at zero time. The essential oil concentrations used were: 0.0 mg/mL (Control), 0.25 mg/mL (MIC_25_), 0.5 mg/mL (MIC_50_) and 2.0 mg/mL (MFC).

Based on the results of the *B. morelensis* essential oil effect on *C. albicans* survival curve, it was observed that a slight population decline (MIC_50_ and MFC) was observed at 12 h compared with the control (Figure 1).

**Figure 2:**
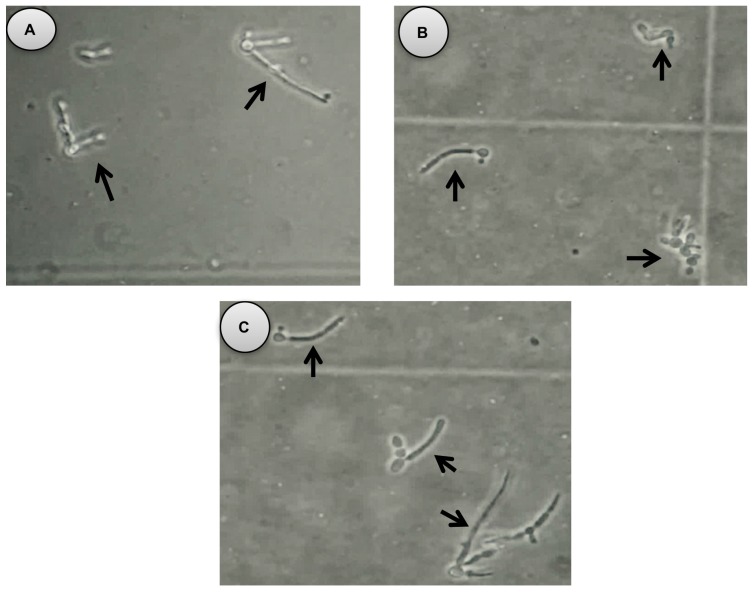
Germ tube growth after two hours of activation with fetal bovine serum with addition of (**A**) Control without essential oil; (**B**) Essential oil at a concentration of 8.0 mg/mL, which resulted in a decreased ability of the yeast to develop a germ tube; (**C**) Essential oil at a concentration of 0.5 mg/mL, with many yeast having normal germ tube growth, but some having poor growth. Arrows indicate the germ tube growth. Microphotographs were taken at 40×.

**Figure 3:**
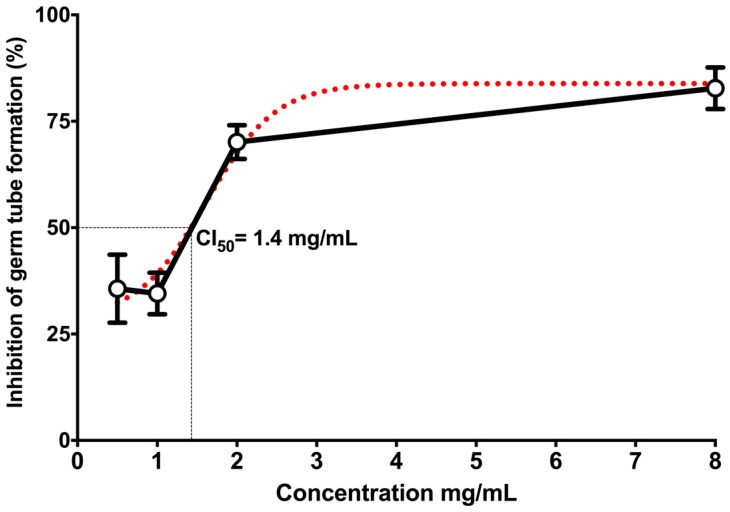
Effect of essential oil from *B. morelensis* concentration on germ tube growth of *C. albicans* 14065. Black line: experimental data; red dotted line: predicted values.

In terms of the effect of the essential oil on the growth of *C. albicans* (14065) germ tube, 8.0 mg/mL of essential oil inhibited 87.94% of the growth of the germ tube after two hours of testing (Figure 2B), which indicates a CL_50_ of 1.4 mg/mL (Figure 3).

**Figure 4:**
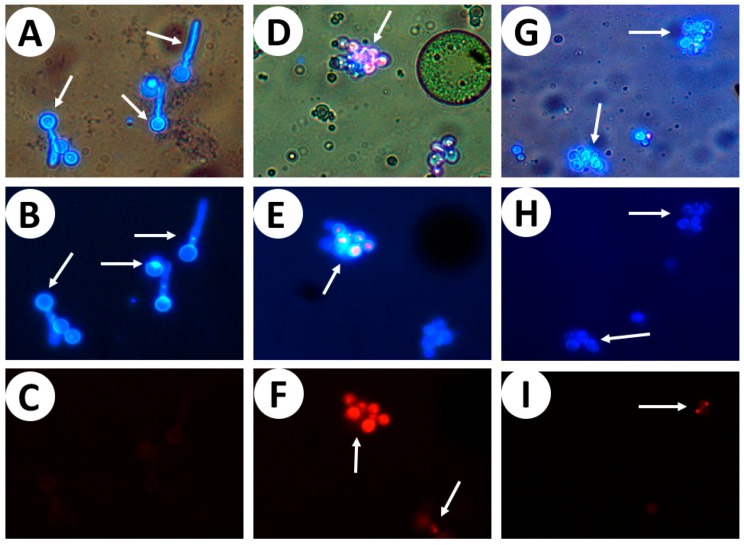
Microphotographs (40×) obtained in fluorescence microscopy and dyed with Calcofluor-white stain ((**B**,**E**,**H**), λ = 360 nm) and propidium iodide ((**C**,**F**,**I**), λ = 536 nm). Visible light (**A**,**D**,**G**). (**A**–**C**) shows the control, while (**D**–**F**) are the yeasts exposed to essential oil (10 mg/mL) for 2 h. (**G**–**I**) show the yeasts exposed to essential oil (2 mg/mL) for 2 h.

The cellular wall of the yeasts of the control tube show a strong blue showing the presence of chitin contained in the cell wall (Figure 4A,B); the cell membrane is intact and the propidium iodide is excluded from viable cells (Figure 4C), since the dye did not access DNA. Nevertheless, 10 mg/mL of the essential oil caused the loss of the integrity of the cell membrane, the propidium iodide easily penetrate the damaged permeable membranes of non-viable cells, and there was a binding of propidium iodide to DNA (Figure 4E,F), which shows the damage in the cellular membrane of *C. albicans* by the essential oil of *B. morelensis*.

**Figure 5:**
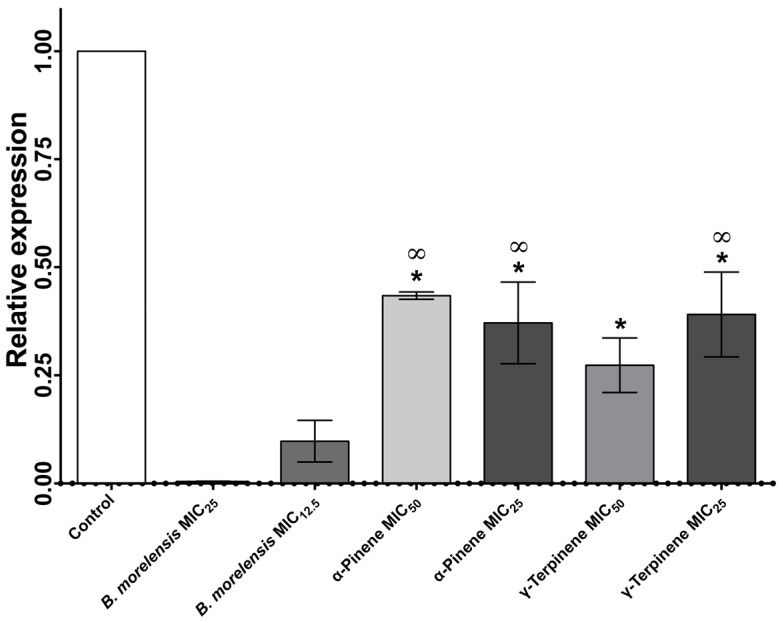
qRT-PCR of the expression of INT1 gene of *C. albicans* exposed to different concentrations of essential oil of *B. morelensis*, α-Pinene and γ-Terpinene. * Significant differences exist regarding the group of *B. morelensis* MIC_25_ (*p* < 0.05). ∞ Significant differences exist regarding the group of *B. morelensis* MIC_12.5_ (*p* < 0.05).

Adherence to host cells and tissues is considered as a key virulence factor of many human fungal pathogens [16]. For this reason, we evaluated the capacity of essential oil of *B. morelensis* to affect the expression of the gene INT1 of *C. albicans*. Figure 5 shows the relative expression analysis of the gene INT1 by qRT-PCR. The essential oil of *B. morelensis* inhibits the expression of INT1, at the MIC_12.5_, at which point there was 10% of the expression. With regards to both pure compounds used at different concentrations, 30–45% expression was observed, which means that the combination of these compounds in the essential oil, increases the independent effect on INT1 expression levels.

## 3. Discussion

By GC–MS analysis, 13 compounds constituting the essential oil of *B. morelensis*, were obtained. The most abundant was γ-Terpinene (65.46%), followed by β-Phellandrene (18.27%). Carrera et al. [12] performed an analysis of the essential oil of *B. morelensis* collected in August and September 2012. They reported 23 compounds, of which 13 are present in the essential oil in this research. The compounds present can be directly related to the season in which samples were collected. Many factors can affect the chemical composition of the essential oils, such as, geographic location of the plants, surrounding climate, seasonal variations, stress during growth, plant ecotype or variety, plant nutrition and application of fertilizers [17].

Zengin and Basal [18], mentioned that the number of double bonds in a structure and the acyclic, monocyclic and/or bicyclic structure has no significant influence on the antimicrobial activity of monoterpenes, although a higher inhibitory activity is seen in aromatic compounds. As the lipophilic character of the membrane allows it to expand, this can result in increased membrane fluidity and permeability, disturbance of membrane-embedded proteins, inhibition of respiration, and alteration of ion transport processes [18]. The α-Pinene has been demonstrated to act on cell integrity, inhibiting respiration and ion transport processes, in addition to increasing membrane permeability in *C. albicans* [19,20,21]. On the other hand, the compounds making up a larger proportion of the essential oil are not necessarily responsible for the majority of the total activity. Therefore, this activity could be attributed to the presence of minor components, such as α-Pinene and β-Pinene (2.85% and 2.30%).

The essential oil of *B. morelensis* had effects on all *Candida* strains tested. The essential oil contains some compounds that induce altered cell permeability by insertion between the fatty acid chains of the phospholipids of the membrane, causing an increase in permeability and fluidity of the same [22,23]. Probably another mechanism of action of essential oils in fungi involves the inhibition of ergosterol synthesis. This is a triterpene present in the fungal membrane, and thus, inhibition would interrupt the formation of the membrane [24,25,26,27].

The essential oil has a fungistatic effect on the survival curve of *C. albicans*. It should be noted that in the case of azoles 20% yeast growth is allowed during the 48-h incubation [28]. It is important to observe that after 12 h of treatment the concentration of MIC_50_ showed a decrease in the fungi population. Nevertheless, the increase of the fungi population with the FMC at 24 h was observed, this is probably due to trailing growth that is observed in the *Candida* species [29]. It is necessary to emphasize that the use of antifungals has been registered from the 1950s [30]. Since this time the development of antifungals against *C. albicans* always has been a priority for the scientific community. However, the prolonged management of candidiasis using antifungals might cause the development of drug-resistant candidiasis [12,13,14]. An alternative has involved the search for new treatments in medicinal plants.

The yeast-to-hyphal transition of *C. albicans* is linked to a number of properties, which is important for its interactions with the host. These properties include adhesion to epithelial and endothelial cells, primary and intercellular invasion via induced endocytosis, active penetration and escape of the phagocytes and immune evasion [31,32]. This transition plays a fundamental role in the virulence and pathogenicity [33,34]. According to the above information, it is important to assess whether the essential oil of *B. morelensis* has the ability to inhibit the growth of *C. albicans* [35]. For this reason, the inhibition of germ tube was evaluated which obtained an CL_50_ of 1.4 mg/mL. It is noteworthy that this the first study about examining the effect of the *B. morelensis* essential oil on the inhibition of the germ tube of the *C. albicans*. This potential pharmacological target is important since the essential oil is acting on an important process in the morpho-transformation of *C. albicans* [21]. Furthermore, this essential oil also might act at the level of the synthesis occurring at the activation of glucan synthase, which is associated with the extensive synthesis of cell wall during the formation of hyphae [36]. This characteristic could suggest a new antifungal which is more selective towards the infecting yeast than towards the host cells [21] so we can’t ignore this pathway inhibition of *C. albicans*.

It is important to indicate that the cell wall structure is dynamic and can adapt to different physiological states or environmental conditions [37]. It must provide the cell with sufficient mechanical strength to withstand changes in osmotic pressure by the environment [38]. Using the Calcofluor white stain, it can be seen the presence of chitin contained in the yeast cell wall. The integrity of cell membrane was lost using the concentration of 10 mg/mL of essential oil, propidium iodide is able to be inserted in the genetic material, which suggests that the essential oil also damages the cell membrane. Furthermore, it is known that the essential oils have the ability to damage the membrane mitochondrial [39], which might be one mechanism of action of the *B. morelensis* essential oil. This is very important because the damage generated in the cell membrane of *C. albicans* causes a profound effect on the growth and morphology of the fungal cell. This often renders it susceptible to lysis and death [38].

qRT-PCR allowed us to show the effect of the essential oil of *B. morelensis* on the expression of INT1, compared to different concentrations of pure compounds (α-Pinene and γ-Terpinene). After 24 h of interactions, we observed that the expression of the INT1 was drastically inhibited in the samples treated with the essential oil of *B. morelensis*. There were significant differences in the results of the essential oil of *B. morelensis* and the pure compounds. These results suggest a possible synergism between the compounds that constitute the essential oil of *B. morelensis*. Nevertheless, we must emphasizes that the essential oil alters the expression of the gene that codifies the integrin INT1p. This is very important since the integrins are known to be important in the adhesion of *C. albicans* [40]. It has been mentioned that INT1p is an integrin involved in the adhesion of *C. albicans* to the host epithelial tissue [41]. Strains of *C. albicans* with knock-out INT1 genes are unable to adhere [7]. This is another possible mechanism of action of the essential oil of *B. morelensis*.

These factors of virulence include host recognition, which enables the pathogen to bind to the host cells and proteins, and adherence to host surfaces [5,42]. The growth of the germ tube [3,32] and the dimorphism between others are essential in the development of the infection caused by this yeast. Nevertheless, the use of the *B. morelensis* essential oil inhibited several of these factors, this ensure the success of the treatment. Therefore, the *B. morelensis* essential oil may be a new treatment for candidiasis.

## 4. Material and Methods

### 4.1. Collection and Identification of B. morelensis

The *B. morelensis* stems were collected in April 2011 in San Rafael, Coxcatlan, Puebla, while the botanical authentication of the specimen was done by M. C. Maria Edith Lopez Villafranco (curator at the IZTA Herbarium). Specimens were deposited in the herbarium IZTA at the Facultad de Estudios Superiores Iztacala (voucher No. 2412 IZTA). 

The specimens were collected in the field with permission from the “Secretaria de Medio Ambiente y Recursos Naturales” (SGPA/DGVS/1266).

The collection site was San Rafael, which is a village in the municipality of Coxcatlan. This village is located southeast of the Tehuacan-Cuicatlan Valley at coordinates of 18°12′–18°14′ N and 97°07′–97°09′ W, which is 957 m above the sea level [43]. The climate is dry or arid with summer rains and a mean temperature of 22 °C [44].

### 4.2. Essential Oil Extraction

Essential oil from *B. morelensis* was obtained by the hydro-distillation method. The distillation equipment consisted of a round-bottomed flask of 1000 mL with a heating mantle (SEV-Prendo, MC301-9, Mexico City, Mexico) which was attached to a double pass condenser (designed by the Laboratory of Pharmacognosy at the FES Iztacala, UNAM, Tlalnepantla, Estado de Mexico, Mexico). This condenser was coupled to a cold-water circulator with controlled temperature. The essential oil was separated from the aqueous phase by density differences.

### 4.3. Essential Oil Chemical Composition

The analysis of the essential oil from *B. morelensis* was performed by GC–MS (Gas Chromatography–Mass Spectrometry) in a model 6850 chromatograph coupled to a mass spectrometer model 5975C, Agilent Technologies. The analysis was performed using a HP–5MS column Agilent Technologies, which had a length of 30 m, internal diameter of 0.25 mm and film of 0.25 microns. The type of injection was split, and the volume of sample used was 1 μL. The separation conditions were as follows: an initial temperature of 70 °C for two min followed by two increasing heating ramps. The first increasing heart ramp was at 20 °C per min up to 230 °C, while the second was in ramp at 8 °C per min up to 280 °C and held for five min, using a carrier gas of Helium. The total analysis time was 21.25 min. The detected mass range was 35–750 *m*/*z*, the sample was ionized by electron impact at 70 eV, and the temperature reached by the ionization source was 230 °C. The identification of the compounds was carried out by comparison with the database version 8.0 NIST library.

### 4.4. Fungal Strains

The extract was tested on four species of *C. albicans: C. albicans* ATCC 14065, *C. albicans* ATCC 32354, *C. albicans* ^1^ (donated by the Laboratory of Clinical Analysis of the FES-CUSI Iztacala), and *C. albicans* CDBB-L-1003.

### 4.5. Anti-Candida Assays

The anti-*Candida* activity was measured by the disk diffusion method [45]. The microorganisms were cultured at 36 °C for 48 h in 10 mL of Sabouraud broth (Difco, Tlalnepantla, Estado de Mexico, Mexico). Cultures were adjusted to turbidity comparable with that of McFarland no. 0.5 standard with sterile saline solution. Petri dishes containing PDA agar (Bioxon, Tlalnepantla, Estado de Mexico, Mexico) were impregnated with these microbial suspensions. Filter paper disks (Whatman No. 5) with a diameter of 5 mm were impregnated either with 5 μL of the essential oil, α-Pinene (5 μL) or γ-Terpinene (5 μL) (Sigma-Aldrich, St. Louis, MO, USA). Disks impregnated with Nystatin (25 μg) were used as positive controls. The plates were incubated overnight at 36 °C, and the diameter of growth inhibition zones (mm) was measured. Each experiment was repeated at least three times.

For quantitative assays, the broth dilution technique was used. An inoculum (4 µL) of 48 h growth was used. This inoculum was placed into microcentrifuge tubes (1.5 mL) with 396 µL of Sabouraud broth. A total of 4 µL of Tween 80 (Sigma-Aldrich, St. Louis, MO, USA) (0.01% *v*/*v*) was then added, and finally the necessary amount of essential oil (4.0–0.065 mg/mL) or pure compounds (16.0–2.0 mg/mL) was added for each of the tested concentrations. A total of 130 µL of this solution, was then taken and placed in microcentrifuge tubes with a smaller capacity (0.6 mL). This was performed in triplicate. After the mixtures with the oil and pure compounds were incubated at 36 °C for 24 h, a sample was taken and grown in the petri dish for counting Colony Forming Units (CFU). Furthermore, 24 h after the concentration of Minimum Fungicidal Concentration (MFC), Fungicidal concentration at 75% (MIC_75_), Fungicidal concentration at 50% (MIC_50_) and Fungicidal concentration at 25% (MIC_25_) were determined in mg/mL.

### 4.6. Survival Curve Assay

A tube for at least one of each concentration to test (preferably the correspondent concentrations to MFC, MIC_50_ and MIC_25_), were prepared with samples taken at 0, 6, 12, 24 and 48 h. A tube without extracts served as the development control. The inoculum with approximately 1 × 105 yeast/mL was prepared in a test tube with 10 mL of Sabouraud broth (this fungal concentration is achieved within 48 h of incubation).

### 4.7. Germ Tube Formation Assay

The germ tube formation assay was conducted in order to study the effect of essential oil on the yeast-to-hyphal transition of *C. albicans* 14065. Different concentrations (2, 0.5 and 0.25 mg/mL) of essential oil was diluted in fetal bovine serum (500 µL) and tween 80 (0.01% *v*/*v*). A total of 4 µL of an inoculum of 5 × 10^6^ CFU/mL was added and the cells were incubated at 36 °C for 3 h. After this, the germinated cells were counted (control germ tube). We classified test tubes as germinated if they had a level of germ tubes three times higher than the original yeast. The negative control of tween 80 (0.01% *v*/*v*) was used. The assay was done by triplicate and were reported as percentage inhibition.

### 4.8. Cell Wall Integrity

Calcofluor-white (Sigma-Aldrich, St. Louis, MO, USA) and propidium iodide (Sigma-Aldrich, St. Louis, MO, USA) dyes were used to evaluated the activity of the essential oil on the cell wall integrity of the *C. albicans*. Concentrations of 2 and 10 mg/mL of the essential oil was used, while a tube without treatment with the essential oil was used as a control.

### 4.9. RNA Extraction and cDNA Synthesis 

Total RNA was isolated from suspensions with 1 × 10^5^ yeast/mL containing different concentrations of *B. morelensis* essential oil. This was prepared as explained above using the AllPrep kit (Qiagen, Hilden, Germany) according to the manufacturer’s operating instructions for yeast cells. Genomic DNA (gDNA) was removed from purified RNA by using TURBO DNAse (Ambion, Carlsbad, CA, USA) according to manufacturer’s instructions. RNA quality was checked by agarose gel electrophoresis at 80 V for 40 min and concentration was measured for purity estimation using the fluorometer (Thermo, Waltham, MA, USA). Single-stranded cDNA was synthesized using SuperScript^®^ III Reverse Transcriptase Kit (Thermo, Waltham, MA, USA) with oligo-dT.

### 4.10. Quantitative Real-Time Polymerase Chain Reaction (qRT-PCR) 

Synthesized cDNA was used to amplify *Candida albicans* INT1 gene with the primers and conditions established by Lim and Li [44]. B-actin was used as a housekeeping gene to normalize the expression [46]. The level expression of INT1 gene was calculated according to E = Peff(−ΔCt), where Peff is the primer efficiency calculated using LinRegPCR [47]. Fold changes were calculated between the ratio expressions of all conditions analyzed for two biological replicates.

### 4.11. Statistical Analysis

All experiments were performed in triplicate. The mean and standard deviation of three experiments were determined. Analysis of the data was done using the one-way analysis of variance (ANOVA) with a Tukey-Kramer Multiple Comparison post hoc test (*p* < 0.05) using GraphPad Prism 7 software. Inhibitory concentration 50 (IC_50_), in the germ tube growth assay, by interpolation in the graph from the inhibition of germ tube formation (%) versus the concentration in mg/mL of the essential oil and through a logarithmic regression analysis with GraphPad Prism 7 software.

## 5. Conclusions

The essential oil of *B. morelensis* has more activity than the two pure compounds. This oil shows capacity for the inhibition of the growth of the germ tube. This also caused disruption of the cell membrane. The essential oil reduces the transcription of INT1, showing significantly differences between the oil and the pure compounds. The results of this investigation show the anti-*Candida* activity of the essential oil.

## Figures and Tables

**Table 1:** Composition of essential oil of *Bursera morelensis* identified by GC–MS (Gas Chromatography–Mass Spectrometry) analysis.

Components	Retention Time	Abundance (%)
Thujane	4.924	0.15
α-Pinene	5.052	2.85
α-Phellandrene	5.453	1.58
β-Pinene	5.541	2.30
β-Phellandrene	5.814	18.27
γ-Terpinene	6.078	65.46
Isobutylbenzene	7.714	0.32
Cyclohexane	8.339	0.44
4-butan-2-yl-2,3-dihydrofuran	8.531	0.39
2-Acetylcyclopentanone	8.651	0.46
Caryophyllene	9.357	5.13
α-Caryophyllene	9.597	0.27
Caryophyllene oxide	10.479	0.34

**Table 2:** Anti-*Candida* activity of essential oil from *B. morelensis*, α-Pinene and γ-Terpinene.

Strains	*B. morelensis*	α-Pinene	γ-Terpinene	Positive Control (Nystatin)
*C. albicans* ^1^	10.0 ± 1.00	9.7 ± 0.58	7.0 ± 0.00	18.0 ± 1.00
*C. albicans* 14065	11.8 ± 0.76	11.3 ± 0.58	6.33 ± 0.58	19.67 ± 0.50
*C. albicans* 32354	11.3 ± 0.58	11.3 ± 1.15	7.0 ± 0.00	22.0 ± 2.00
*C. albicans* CDBB-L-1003	11.2 ± 0.29	8.3 ± 0.58	6.0 ± 0.00	22.0 ± 1.00

Inhibition halos measured in millimeters. ^1^ Donated by the Clinical Laboratory of FES-Iztacala. Filter paper disks were impregnated either with 5 μL of essential oil, α-Pinene (5 μL) and γ-Terpinene (5 μL).

**Table 3:** MIC (Minimal Inhibitory Concentration) and MFC (Minimum Fungicidal Concentration) of essential oil and pure compounds.

Strains	*B. morelensis*				α-Pinene				γ-Terpinene			
	MFC	MIC_75_	MIC_50_	MIC_25_	MFC	MIC_75_	MIC_50_	MIC_25_	MFC	MIC_75_	MIC_50_	MIC_25_
*C. albicans* ^1^	2.0	0.25	0.125	0.062	4.0	1.0	0.5	0.125	16.0	12.0	6.0	4.0
*C. albicans* 14065	2.0	1.0	0.5	0.125	4.0	2.0	0.5	0.125	16.0	14.0	10.0	8.0
*C. albicans* 32354	2.0	0.5	0.125	0.062	4.0	1.0	0.5	0.065	16.0	10.0	6.0	2.0
*C. albicans* CDBB-L-1003	2.0	0.25	0.125	0.062	2.0	1.0	0.5	0.25	12.0	10.0	8.0	6.0

Concentrations in mg/mL. ^1^ Donated by the Clinical Laboratory of FES-Iztacala.
